# Educating the blind brain: a panorama of neural bases of vision and of training programs in organic neurovisual deficits

**DOI:** 10.3389/fnint.2014.00089

**Published:** 2014-12-05

**Authors:** Olivier A. Coubard, Marika Urbanski, Clémence Bourlon, Marie Gaumet

**Affiliations:** ^1^The Neuropsychological Laboratory, CNS-FedParis, France; ^2^Laboratoire Psychologie de la Perception, UMR 8242 CNRS-Université Paris DescartesParis, France; ^3^Service de Médecine et de Réadaptation Gériatrique et Neurologique, Hôpitaux de Saint-MauriceSaint-Maurice, France; ^4^Institut du Cerveau et de la Moelle Epinière (ICM), Sorbonne Universités, Université Pierre et Marie Curie UM 75, Inserm U 1127, CNRS UMR 7225Paris, France; ^5^Service de Médecine et de Réadaptation, Clinique Les Trois SoleilsBoissise-le-Roi, France

**Keywords:** binocular vision, eye movements, visual pathways, neurovisual disorders, visual rehabilitation

## Abstract

Vision is a complex function, which is achieved by movements of the eyes to properly foveate targets at any location in 3D space and to continuously refresh neural information in the different visual pathways. The visual system involves five main routes originating in the retinas but varying in their destination within the brain: the occipital cortex, but also the superior colliculus (SC), the pretectum, the supra-chiasmatic nucleus, the nucleus of the optic tract and terminal dorsal, medial and lateral nuclei. Visual pathway architecture obeys systematization in sagittal and transversal planes so that visual information from left/right and upper/lower hemi-retinas, corresponding respectively to right/left and lower/upper visual fields, is processed ipsilaterally and ipsialtitudinally to hemi-retinas in left/right hemispheres and upper/lower fibers. Organic neurovisual deficits may occur at any level of this circuitry from the optic nerve to subcortical and cortical destinations, resulting in low or high-level visual deficits. In this didactic review article, we provide a panorama of the neural bases of eye movements and visual systems, and of related neurovisual deficits. Additionally, we briefly review the different schools of rehabilitation of organic neurovisual deficits, and show that whatever the emphasis is put on action or perception, benefits may be observed at both motor and perceptual levels. Given the extent of its neural bases in the brain, vision in its motor and perceptual aspects is also a useful tool to assess and modulate central nervous system (CNS) in general.

## Introduction

Born in Canada and USA at the beginning of the 20th century and in France in the 1950s under the impulsion of Henri Hécaen, neuropsychology examines the relationship between cognitive activity (attention, perception, gesture, memory, language, etc.) and corresponding cerebral condition (the different areas of the central nervous system—CNS—from spinal cord to cortex). At the frontiers of neurology, psychology and psychiatry, neuropsychology explores how brain and function may be correlated in brain-damaged patients or by using functional brain imaging (Diffusion Tensor Imaging, DTI; functional Magnetic Resonance Imaging, fMRI; Positron Emission Tomography, PET; Single Photon Emission Computed Tomography, SPECT). In close collaboration with neurologists and psychiatrists, neuropsychologists assess and rehabilitate brain-damaged patients by acting onto sensory, motor, cognitive and emotional spheres. As a branch of neuropsychology, visual neuropsychology specifically studies vision in its sensory, motor, cognitive and emotional dimensions. As such, visual neuropsychology focuses on the nervous part of visual function, from retina to the multiple areas of the brain it involves. Given that 60% of the brain participates in vision (Orban et al., 2004; Orban, 2007), the extent of this study field is wide, from the most elementary visual functions (visual acuity, contrast sensitivity, visual field, color, depth, movement or visuo-spatial perception) to the most complex ones (object and face identification, perception of scenes and of emotions, written language processing, action-perception interaction, etc.). Similarly, neurovisual pathologies extend from low-level (partial or complete loss of visual field, achromatopsia, astereopsia, akinetopsia, etc.) to high-level disorders (visual agnosias, prosopagnosia, visual alexia, Balint syndrome, etc.). In this didactic review for both experts and novices, we provide a panorama of the neural bases of vision in its motor and perceptual aspects: eye movements and visual systems, respectively. Based on this knowledge, we briefly review the different damages that can occur in the visual systems, before overviewing the different rehabilitation schools of visual neuropsychology, which were developed in Europe and USA since the 1970s. The present review belongs to a Frontiers in Integrative Neuroscience e-book containing eighteen other contributions, and as such will offer reference throughout the text to those articles related to either Eye movements or Visual Perception or Visual training programs (Coubard, in press).

## From action to perception

**Figure 1:**
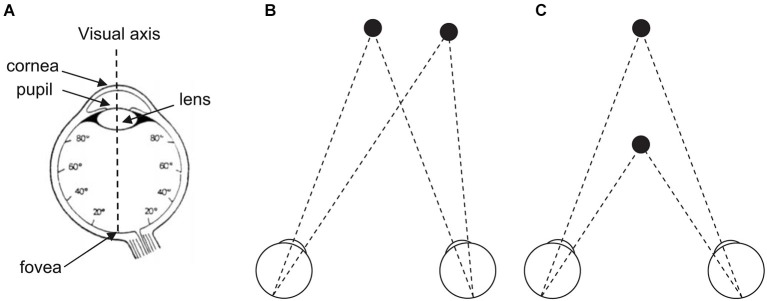
**(A)** The human eye and its visual axis. The fovea (1° to 2° of visual angle) contains high density of cones but no rod. Cone concentration decreases with increasing distance to the fovea and stabilizes for the rest of the retina. Rod concentration reaches its maximum at 20° of retinal eccentricity than decreases until the peripheral limit of the retina. **(B)** The two eyes move in direction thanks to a step (saccade) or smooth (pursuit) movement to foveate a target in eccentricity (to the left, to the right, up, down, or in any oblique direction). **(C)** The two eyes move in depth thanks to a step or smooth (vergence) movement to foveate a target in distance (at close or at far). **(A)** Adapted from Bagot (1999, p. 128, Figure 35) (© O.A. Coubard, with permission); **(B,C)** From O.A. Coubard.

“In the beginning was the act” (Von Goethe, 1808–1832/2014). In line with von Goethe, we point out in this review that vision is first and foremost action. The reason why the eyes move is twofold. First they move as direct consequence of retina morphophysiology (see Figure 1A). Only the fovea containing a high density of cones allows humans to perceive visual stimuli with high acuity, while the rest of the retina containing less cones but high density of rods perceives blur. For that reason, the eyes have to move to foveate visual stimuli in eccentricity or in depth. Second the eyes move as visual perception is impossible as soon as movement is absent, which has been demonstrated different ways since the seminal work by Yarbus (1967). Indeed when fixational eye movements are suppressed and the visual stimulus stabilized on the retina, perception just vanishes in a few seconds. This is due to the fact that one function of fixational eye movements, among other functions, is to continuously refresh neural activity in visual pathways (for a review see Martinez-Conde et al., 2013).

To visually explore the world, humans make a variety of eye movements. Saccades are step movements in direction to foveate targets at different locations, while smooth pursuit aims at following a moving target in direction (see Figure 1B). Vergence is a movement of the eyes in depth, which can be step as saccades or smooth as pursuit (see Figure 1C). For an original research on functional brain imaging of vergence eye movements, see in the present e-book the article by Alvarez et al. (2014). Eye movements can be not only volitional but also automatic in response to sudden stimuli in any modality (reflexive eye movements), to stabilize images on the retina during head movements (vestibulo-ocular reflex), or to gaze moving visual patterns (optokinetic reflex). Even when fixating a stationary point, the eyes are never at rest but move through micromovements, tremor, drifts, microsaccades, which are critical for vision as mentioned above (for reviews see Carpenter, 1988; Leigh and Zee, 1999; Coubard, 2011; Martinez-Conde et al., 2013). For a review on fixational eye movements and binocular vision, see in the present e-book the article by Otero-Millan et al. (2014).

**Figure 2:**
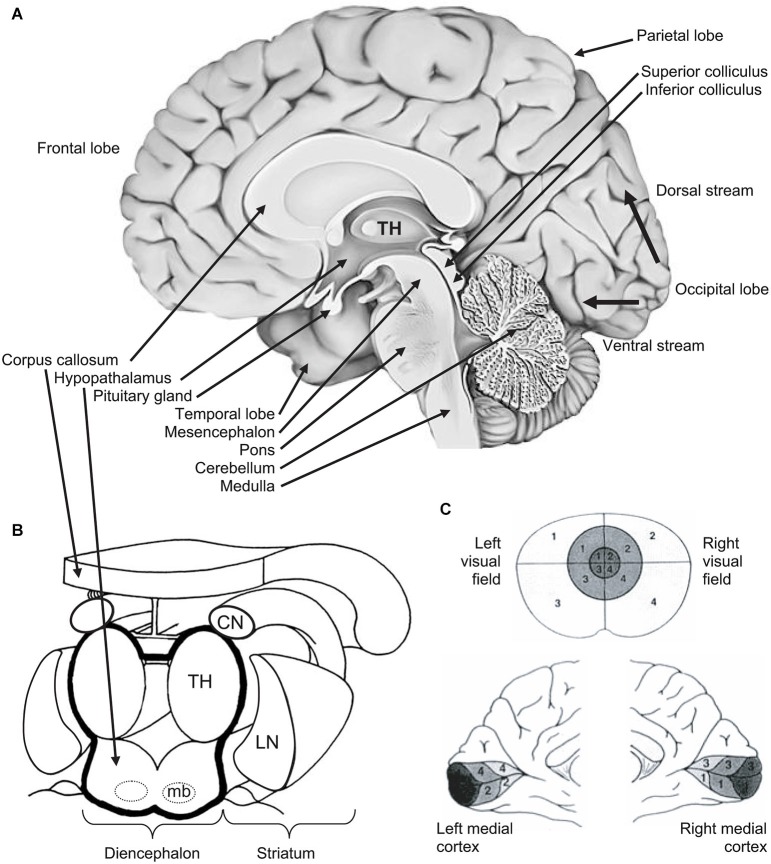
**(A)** Medial view of right hemisphere showing the brainstem and its three levels (from bottom to top: medulla, pons, mesencephalon), the diencephalon, the telencephalon and its four hemispheric lobes (clockwise: frontal, parietal, occipital, temporal). From the primary visual area (V1) in the occipital cortex, thin black arrows show the ventral (down) stream and the dorsal (up) streams. **(B)** 3D view of diencephalon and striatum. The diencephalon is delineated by a thick black line. The striatum (CN: caudate nucleus; LN: lentiform nucleus) is in close vicinity of the diencephalon. **(C)** Retinotopy of the retino-occipital visual pathway: organization of projections of the visual field (upper part) in the occipital cortex where the primary visual cortex (or V1) is spread around the calcarine sulcus (medial views in the lower part). Information from central visual field (foveal vision—the darkest in the figure) is projected and over-represented in the posterior part of occipital cortex. Other notations: mb: mammillary bodies; TH: thalamus. From O.A. Coubard (© O.A. Coubard, with permission).

**Figure 3:**
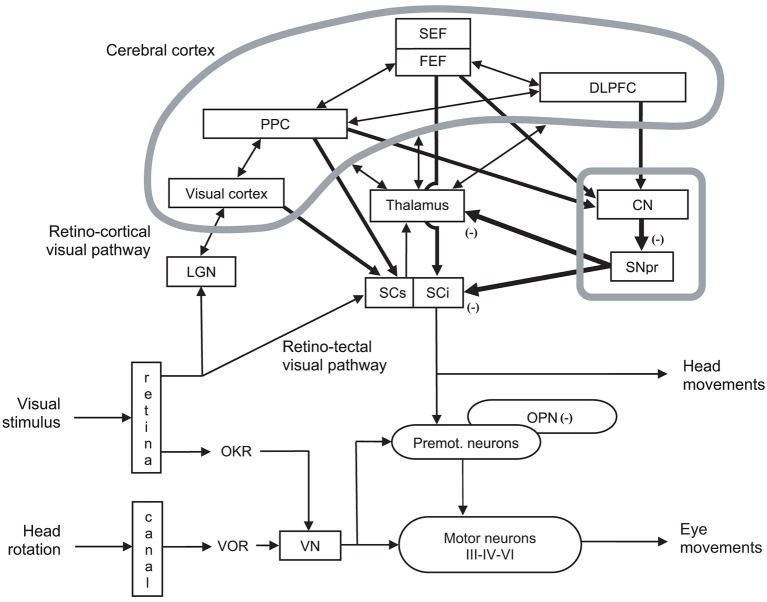
**Organization of cerebral structures involved in the control of eye movements, specifically of ocular saccades**. The visual stimulus activates the retino-cortical visual pathway (LGN: lateral geniculate nucleus; visual cortex), which activates associative cortex (PPC: posterior parietal cortex), as well as cortical (SEF: supplementary eye field; FEF: frontal eye field) and subcortical areas (CN: caudate nucleus; SNpr: substantia nigra pars reticulata) involved in eye movement control by acting onto thalamus and the motor part (intermediate layer) of superior colliculus (SCi). The visual stimulus activates in parallel the retino-tectal visual pathway through the sensory part (superficial layer) of superior colliculus (SCs), which directly activates SCi. In fine, eye movements are triggered by premotor (Premot.), which are under the inhibitory control of omnipause neurons (OPN). Premotor neurons activate motor neurons of oculomotor (III), trochlear (IV) and abducens (VI) nerves. Eye movements also interact with head movement (not developed). Other notations: DLPFC: dorsolateral prefrontal cortex; OKR: optokinetic response; VN: vestibular neuron; VOR: vestibulo-ocular reflex. Adapted from Hikosaka et al. (2000, p. 956, Figure 3).

For didactic purpose, Figure 2 illustrates the brain and its subcortical and cortical architecture. Eye movements are performed thanks to extraocular muscles, which are controlled by a cascade of physiological mechanisms (Hikosaka and Isoda, 2010; see Figures 2, 3). At the lowest level, extraocular muscles are directed by motoneurons: the lateral rectus is innervated by abducens nerve (VI), the superior oblique by trochlear nerve (IV), and other muscles (medial rectus, superior and inferior recti, inferior oblique, as well as intrinsic muscles) by the oculomotor nerve (III). Motoneurons are themselves directed by premotor or burst neurons, which generate three patterns of innervation: the pulse is the velocity to rotate the eye; the step is the position to maintain the eye in its new position; the slide, between the pulse and the step, counteracts viscoelastic forces of the oculomotor muscles and globe in the orbit (for reviews see Scudder et al., 2002; Coubard, 2013; see Figure 3). Premotor neurons for horizontal saccades are located in the paramedian pontine reticular formation (PPRF), the medullary reticular formation (medRF), the nucleus prepositus hypoglossi (NPH), and the medial vestibular nucleus (MVN). PPRF and medRF premotor neurons provide the pulse force, whereas the step force is achieved by bilateral NPH and adjacent MVN. PPRF premotor neurons are excitatory and contact ipsilateral motoneurons. Premotor neurons of medRF are inhibitory contacting contralateral motoneurons (Fuchs et al., 1985; Langer et al., 1986; Moschovakis et al., 1996; Scudder et al., 2002). Premotor neurons for vertical saccades are located in the rostral interstitial nucleus of the medial longitudinal fasciculus (riMLF; Büttner-Ennever and Büttner, 1978; King and Fuchs, 1979), while those for vergence have been found in the mesencephalic reticular formation (MRF; Mays, 1984; Judge and Cumming, 1986; Mays et al., 1986). Premotor neurons of any type of eye movements in any direction are under common inhibitory control of so-called omnipause neurons (OPN) confined in the nucleus raphe interpositus in the brainstem (Büttner-Ennever et al., 1988; see Figures 2, 3).

At a higher level, the premotor circuitry is controlled by the superior colliculus (SC), which can elicit on its own reflexive eye movements (Schiller et al., 1987; Chaturvedi and van Gisbergen, 1999), i.e., eye movements with reaction time from 60 ms in monkeys (Fischer and Boch, 1983) and 80 ms in humans (Fischer and Ramsperger, 1984). This is possible thanks to the direct retinotectal pathway to the superficial, visual layers of the SC and then direct activation of SC motor neurons by SC visual neurons, probably through the interlaminar connection between its superficial and intermediate layers (Mooney et al., 1988; Isa and Kobayashi, 2004). SC movement-related neurons located in its caudal pole activate premotor neurons (Moschovakis et al., 1996; Chaturvedi and van Gisbergen, 1999), while SC fixation neurons in the rostral pole prevent premotor neurons through OPNs (Munoz and Wurtz, 1993a,b; Chaturvedi and Van Gisbergen, 2000; see Figures 2, 3). Whether the SC neurons in the rostral pole play the same role but simply for small movement amplitudes (Hafed and Krauzlis, 2008), or are a separate fixation population through their connections to the OPNs (Munoz and Wurtz, 1993a,b) remains an open question.

At the cortical level, the SC is controlled by the posterior parietal cortex (PPC) and more specifically parietal eye field (PEF) for triggering reflexive movements (Paré and Wurtz, 2001) and the frontal eye field (FEF) for intentional movements (Schall et al., 2011), while the supplementary eye field (SEF) plays a role in movement preparing (Nachev et al., 2008). PEF is also involved in visual attention and in spatial updating of visual information (Pierrot-Deseilligny et al., 2004). For a review on the role of FEF in eye movements, see in the present e-book the article by Percheron, François and Pouget (What makes a frontal eye field area the frontal eye field area?, under review). The inhibitory control of SC is achieved by the substantia nigra pars reticulata (SNpr), which is itself gated by subcortical structures such as the dorsal striatum (Hikosaka et al., 2000). The SC also receives inhibition from the dorsolateral prefrontal cortex (DLPFC) through a direct prefrontotectal tract (Goldman and Nauta, 1976; Leichnetz et al., 1981; Gaymard et al., 2003). For a review on the role of DLPFC in eye movements, see in the present e-book the article by Funahashi (2014). Finally, cerebellum maintains accuracy (Prsa and Thier, 2011; see Figures 2, 3).

In summary, the subtle cascade of physiological excitatory and inhibitory mechanisms, allowing eye movements to be performed with appropriate timing and precision, reveals the complexity of oculomotor control. For a review on decisional aspects of eye movements, see in the present e-book the article by Noorani (2014). The quality of eye movements and of binocular coordination is a prerequisite for fusion of the object of interest on the two foveas thus ensuring binocular visual perception.

## The seeing brain from eye to cortex

**Figure 4:**
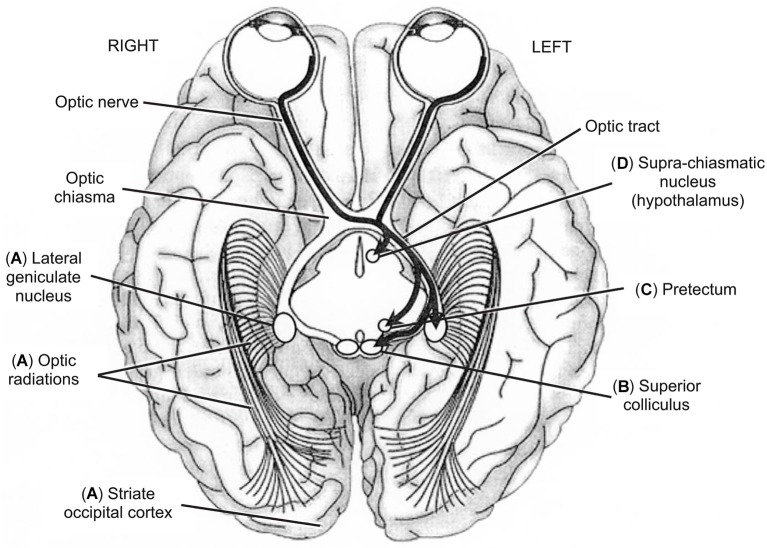
**Inferior view of the brain showing the five main visual pathways, in which left is right and vice-versa**. **(A)** Retino-occipital or retino-cortical visual pathway. **(B)** Retino-collicular or retinotectal visual pathway. **(C)** Retino-pretectal visual pathway. **(D)** Retino-hypothalamic visual pathway. Accessory optic visual pathway is not shown. Thick arrows show the different trajectories of ganglion neurons originating in leftward hemi-retinas (temporal hemi-retina of the left eye and nasal hemi-retina of the right eye). Ganglion neurons originating in rightward hemi-retinas exhibit mirror trajectories but are not illustrated here for clarity of display. From O.A. Coubard (© O.A. Coubard, with permission).

Binocular vision is achieved by five main neurovisual systems originating in the retina but varying in their destination within the brain (see Figure 4). Two systems have been widely studied: the retino-occipital or retino-cortical visual pathway (see Figure 4A) and the retino-collicular or retino-tectal visual pathway (see Figure 4B). But there also exist three other systems: the retino-pretectal (see Figure 4C) and the retino-hypothalamic (see Figure 4D) visual pathways, as well as the accessory optic system (AOS) (not illustrated), which play a crucial role in vision though they are less known. The first neuron that is given the information from sensory cells—cones and rods—is the bipolar neuron. Interestingly, bipolar neurons transfer information to the second neuron, the ganglion neuron or retinal ganglion cell (RGC), within the retina. This means that CNS is already present in the peripheral organ for vision, reminding us that the eye is ontogenetically a differentiation of the diencephalon (see Figures 2A,B). This is how authors and artists see in the eye a door directly open to the mind (e.g., Marendaz et al., 2007). On the scientific viewpoint, it will be our rationale for using eye movements and vision as useful indicators of CNS (dys)functioning.

In mammals, RGCs include at least 20 different subtypes and directly target more than 24 brain areas. Each RGC subtype responds to a specific feature in the visual scene, though the function of many of them remains to be elucidated (for a recent review see Dhande and Huberman, 2014).

The retino-occipital visual system is the most evolved in phylogenesis. In this system (see Figure 4A), RGCs are retino-thalamic as they exit the eye through the optic nerve, go through the optic tract to reach the lateral geniculate nucleus (LGN), where they contact the third neuron, the thalamo-cortical neuron. Importantly between the optic nerve and tract, ganglion fibers originating in nasal hemi-retinas cross the optic chiasma whereas those originating in temporal hemi-retinas do not (see Figure 4). Such lateral systematization has consequences that will be described below (see Section The Blind Brain from Optic Neuritis to Neglect). Thalamo-cortical neurons exit LGN, go through the optic radiation or geniculo-calcarine tract to reach the primary visual or occipital striate cortex or V1 or Brodmann’s area 17, where preliminary processing of visual information is achieved (Merigan and Maunsell, 1993; Bullier, 2001; Kaplan, 2003; see Figure 4). The retino-occipital visual system is mainly made of parvocellular neurons (P/X cells in animals), which convey high spatial frequencies allowing fine analysis of the visual scene (Merigan and Maunsell, 1993; Espinosa and Stryker, 2012). The corollary of such sophistication is its slowness as compared to other visual pathways in which visual information is processed faster and earlier but with lower resolution (Bullier, 2001; Kaplan, 2003). Some magnocellular neurons (M/Y cells in animals) are also present but in lower proportion. Recent studies using transgenic labeling of specific RGCs in mouse have demonstrated that LGN contains at least two functional categories of cells: direction selective RGCs (DSGCs) and non-DSGCs in respectively the shell and the core of LGN (Dhande and Huberman, 2014). W-like cells (small diameter) reside in LGN shell where DSGCs terminate, whereas Y-like cells (large diameter) reside in LGN core where alpha RGCs and non-DSGCs terminate, while X cells (medium diameter) are found both in LGN shell an core (Dhande and Huberman, 2014). From V1, the information is sent to extrastriate (i.e., other than V1) cortex: that from parvocellular neurons project mostly to V2, V4, and the occipital-temporal (ventral) stream involved in object identification; that from magnocellular neurons projet mostly to V2, V3, MT, and the occipital-parietal (dorsal) stream involved in object localization, action and action-perception integration (Ungerleider and Mishkin, 1982; Merigan and Maunsell, 1993; Kravitz et al., 2013; see Figure 4). For a review on neural bases of spatial frequency processing in scene perception, see in the present e-book the article by Kauffmann et al. (2014).

Phylogenetically less evolved than the retino-occipital route, other visual systems play nevertheless an important role in vision. Some of ganglion neurons do not reach LGN but fork earlier in direction of visual neurons located in superficial layers of the caudal pole of the SC (Dhande and Huberman, 2014; see Figure 4B). As evoked in Section From Action to Perception, the SC is responsible for the triggering of reflexive eye movements thanks to motor neurons of its intermediate layers (Schiller et al., 1987; Moschovakis et al., 1996). This contingent of ganglion neurons is made up with magnocellular cells and projects also to the pulvinar, to dorsal stream (Berson, 1988), as well as temporal (Sugase et al., 1999) and frontal (Bullier, 2001) areas of the brain. Such magnocellular pathway subserves an expedient but raw estimate of the visual scene by conveying low spatial frequencies (Bullier, 2001; Bar, 2003; Isa and Kobayashi, 2004). Importantly, the retino-collicular visual system is also linked to limbic structures such as the amygdala and the orbitofrontal cortex, which it is able to rapidly activate in response to fearful stimuli (Krolak-Salmon et al., 2004). This particular feature explains how visual stimuli may have emotional effects. Recent advances using genetic marking of RGCs have identified at least four parallel retinotopically complete maps in mouse SC, but it remains unknown whether these different maps are separate or combined within the network of collicular neurons (Dhande and Huberman, 2014).

Intrinsically photosensitive RGCs (ipRGCs) reach neither LGN nor SC but the pretectum, located between the mesencephalon and diencephalon (Dhande and Huberman, 2014; see Figure 4C). Pretectum contains several nuclei: the pretectal nucleus and tegmental nuclei such as interstitial nucleus, preinterstitial nucleus and epithalamic nucleus. The pretectal nucleus receives direct afferences from the retina and indirect ones from LGN and projects to the autonomic nervous system. Fibers of tegmental nuclei of the reticular formation belong to medial longitudinal fasciculus (MLF; Sprague, 1972; Büttner-Ennever and Horn, 1997). Functionally, the pretectum is critical for the pupillary light or photomotor reflex (Clarke et al., 2003).

A contingent of ipRGCs is directed to hypothalamus, specifically to the supra-chiasmatic nucleus, dorsally to the optic chiasma (Dhande and Huberman, 2014; see Figure 4D). Through melanopsin, this visual system projects to the pineal gland, which itself produces melatonin. As such, the retino-hypothalamic pathway regulates numerous behavioral and biological functions as well as circadian rhythms: temperature, wake/sleep, cortisol, reproduction, autonomic and hormonal functions, etc. (Trachtman, 2010). This explains how visual stimuli may have influence on various biological rhythms and functions.

Finally, the AOS (not illustrated) plays a role in head and gaze orientation as well as slow movements, and generate reflexive eye movements that compensate for retinal slip (Brodsky, 2012). AOS consists of On-DSGCs projecting to two brainstem targets: the nucleus of the optic tract and the dorsal terminal nucleus on the one hand, the medial and lateral terminal nuclei on the other, for controlling respectively horizontal and vertical slip compensation (Dhande and Huberman, 2014).

In recent years, brain functional imaging in humans has put forth our understanding of the different visual systems and has allowed researchers to explore new subsystems. This is how specialized visual areas have been discovered, such as the Fusiform Face Area (FFA) for faces (Sergent et al., 1992), the Parahippocampal Place Area (PPA) for navigation (Epstein et al., 1999), the Lateral Occipital Cortex (LOC) for objects and tools (Grill-Spector et al., 2001), the Extrastriate Body Area (EBA) for human body (Downing et al., 2001), or the Visual Word Form Area (VWFA) within the fusiform gyrus for reading (McCandliss et al., 2003; Dehaene and Cohen, 2011). For recent findings on functional brain imaging of visual pathways, see in the present e-book the article by Raz and Levin (2014).

To summarize, five visual systems participate in vision, two of which are widely studied (the retino-occipital and the retino-collicular systems) and three of which are less known (the retino-pretectal, the retino-hypothalamic and the AOS). Taken together, this neurovisual circuitery represents 60% of brain activity in humans (Orban et al., 2004; Orban, 2007). The ubiquity of vision in the CNS explains on the one hand potential impact on vision that may have any brain injury, and reveals on the other hand the importance of vision in the functioning of the CNS in general and how useful vision may be in neuropsychological assessment and rehabilitation.

## The blind brain from optic neuritis to neglect

We now move to the damages that can occur in the neural visual pathways and rendering blind not the eye but the brain. Before entering the core of visual disorders, we remind two physiological properties of the visual system.

**Figure 5:**
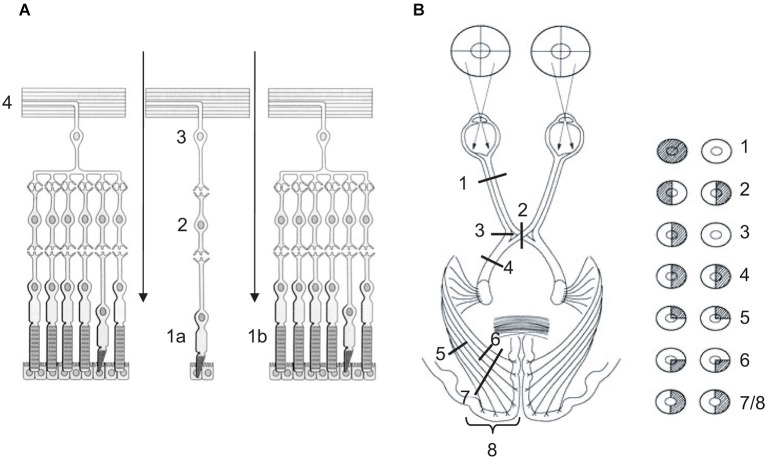
**(A)** Retina or nervous layer of the eye. Light information cross the retina (arrows) to reach sensory cells, cones (1a—fovea) and rods (1b—peripheral retina), which transform light information into neural information. Neural information is transferred to bipolar neurons (2), then to ganglion neurons (3). Fibers of the latter exit the retina (4) and merge into the optic nerve. **(B)** Lateral systematization of the retino-occipital visual pathway. (1) Lesion of optic nerve causes monocular blindness. (2) Lesion of optic chiasma causes bitemporal hemianopia. (3) Unilateral lesion of direct temporal fibers causes monocular nasal blindness. (4) Unilateral lesion of optic tract, or (7) of both lower and upper optic radiations, or (8) of primary visual cortex causes homonymous hemianopia (HH). (5) Unilateral lesion of lower optic radiations causes homonymous superior quadrantanopia. (6) Unilateral lesion of upper optic radiations causes homonymous inferior quadrantanopia. **(A)** Adapted from Bear et al. (1997, p. 224, Figure 9.14) (© O.A. Coubard, with permission); **(B)** Adapted from www.chups.jussieu.fr/ polys /neuro/ semioneuro/ POLY.Chp.3.6.3. html, Figure 8 (© O.A. Coubard, with permission).

A first striking feature of vision is retinal inversion (see Figure 5A). After light information has crossed the cornea, the anterior chamber, the pupil, the lens, the posterior chamber, it has to cross all layers of the retina, as it is inversed, to reach sensory cells. Indeed, cones and rods are opposite to the light for a reason that is hitherto unknown, except that their metabolic and photopigment regeneration requirements need ready access to the choroidal blood supply in the deepness of the retina. Once sensory cells have transformed light into neural information, the latter is transferred to bipolar neuron then to ganglion ones as described above (see Section The Seeing Brain from Eye to Cortex). Due to retinal inversion, ganglion neuron fibers exit the eye making a hole in the retina, the blind spot, to merge into the optic nerve (see Figure 5A). Retinotopy, that is the way information is spatially organized on the retina, is preserved throughout visual pathways and is retrieved particularly in SC and primary visual cortex (Dowling, 1970; Tamraz et al., 1999; Chalupa and Werner, 2003; Podoleanu, 2012; see Figure 2C).

A second striking feature of vision is visual pathways’ systematization in sagittal (left vs. right) and transversal (up vs. down) planes (see Figures 2C, 5B). In the sagittal plane, physiological architecture is such that visual information from left hemi-retinas is processed by the left hemisphere, whereas that from right hemi-retinas remains in the right hemisphere. To achieve this feat, fibers from left (temporal) hemi-retina of left eye remain in the left hemisphere between the optic nerve and tract, while fibers from left (nasal) hemi-retina of the right eye cross the median plane in the chiasma optic to be processed by the left hemisphere. In mirror, fibers from right (temporal) hemi-retina of right eye remain in the right hemisphere, whilst fibers from right (nasal) hemi-retina of left eye cross the median plane in the chiasma optic to be processed by the right hemisphere. Importantly, the fact that information from left/right visual field is processed by right/left hemispheres is only due to the fact that the eyes are globular and, as a direct consequence, visual information is reversed in both sagittal and transversal planes between the physical world and the retinas (Tamraz et al., 1999; Chalupa and Werner, 2003; see Figures 2C, 5B).

In transversal plane, fibers from lower hemi-retinas of the eyes is processed by lower fibers of the optic tracts and radiations, whereas fibers from upper hemi-retinas of the eyes is processed by upper fibers of the optic tracts and radiations. Such transversal distinction has some consequences in terms of damage (see below). As example, optic radiations between LGN and V1 project in such a 3D wave that upper fibers are parietal while lower fibers are temporal. Once again, only because of eyes’ globularity, information from upper visual field is processed in fine in the part of the primary visual cortex that is below the calcarine sulcus, while information from lower visual field is processed in the upper part of V1 above the calcarine fissure (Tamraz et al., 1999; Chalupa and Werner, 2003; see Figures 2C, 5).

These physiological constraints in mind, visual consequences may be more easily inferred from organic damages that can occur at any level of the visual pathways, as exemplified here for the retino-occipital route (see Figure 5B). At the lowest level of visual processing, unilateral lesion of the optic nerve is responsible for monocular blindness. Optic neuritis as observed in multiple sclerosis can result in monocular blindness (Viret et al., 2013; see Figure 5B1). Lesion of optic chiasma, which can be seen in hypophyseal adenoma, results in bitemporal hemianopia: only fibers from nasal retinas decussating in the optic chiasma are injured, resulting in temporal loss of vision bilaterally (Schneider, 1979; Foroozan, 2003; see Figure 5B2). If unilateral lesion concerns only direct fibers of optic chiasma, it results in monocular nasal blindness (see Figure 5B3). Homonymous hemianopia (HH) is inherent in unilateral lesion of either optic tract, or LGN, or optic radiations, or occipital primary visual cortex. HH causes loss of vision in ipsilateral hemi-retinas, thus in contralateral visual field (Grunda et al., 2013; de Haan et al., 2014; see Figures 5B4–7/8). Most common aetiologies for HH are stroke of the posterior cerebral artery (70%), traumatic brain injury (11–14%) and tumors (11%) (Zhang et al., 2006). Visual field defects can be more restricted than HH. Indeed unilateral retrochiasmatric lesion can be restricted to lower (temporal) or upper (parietal) optic radiations, resulting respectively in homonymous superior quadrantanopia (see Figure 5B5) or homonymous inferior quadrantanopia (see Figure 5B6). According to Zhang et al. (2006), 37% and 62% of HH would be complete and incomplete, respectively, with 29% for quadrantanopia. To end, it is worth noting that these low-level visual field defects (HH, quadrantanopia, etc.) have also deleterious impact in high-level functions such as reading or scene perception. As such, hemianopic dyslexia refers to reading deficit inherent in HH without lesion of high-level area related to reading such as VWFA (Schuett et al., 2008). For an original research on eye movements in dyslexia, see in the present e-book the article by Seassau et al. (2014). For scene perception, Perez et al. (2009, 2013) showed that left/right HH differently affect scene detection and categorization.

From V1, damages of the visual pathways yield visual impairments of higher level and complexity. Lesion may be restricted to any part of extrastriate cortex such as V2, V3, V4 or V5/MT. As examples, a specific lesion of V4 results in achromatopsia, the inability to see colors in visual stimuli (Duvelleroy-Hommet et al., 1997; Heywood and Kentridge, 2003), while a specific lesion of V5/MT results in akinetopsia, the inability to see motion in visual stimuli (Zeki, 1991; Nawrot, 2003). At a higher level, organic lesion of the brain may concern either the inferior temporal cortex (the ventral stream or “what” pathway) or PPC (the dorsal stream or “where” or “how” pathway). Ventral stream lesions lead to the constellation of visual agnosias gathering defects of identification, which may specifically concern objects (Konen et al., 2011), faces (Gainotti, 2014), reading (Cohen et al., 2004; Epelbaum et al., 2008), etc. Dorsal stream lesions result in defects in action and visuo-spatial attention such as Balint syndrome (Biotti et al., 2012), or spatial neglect (Corbetta and Shulman, 2011; Harvey and Rossit, 2012).

To end, other neurovisual systems can also be impaired, resulting in specific impairment of the functions related to the retino-collicular (Gaymard et al., 2003), retino-pretectal (Papageorgiou et al., 2009), retino-hypothalamic (Fraser et al., 2012) visual pathways, as well as to AOS (Brodsky, 2012). However these studies are scarce given the low frequency of such focal lesions in humans.

In summary, the different neurovisual systems (retino-occipital, retino-collicular, retino-pretectal, retino-hypothalamic, optic accessory) can be specifically impaired following organic lesion of the brain. Depending on the level at which the lesion occurs in the visual pathway from the optic nerve to their destination within the brain, visual defects concern different levels of visual processing, but low-level deficits may also impact high-level functions.

## Educating the blind brain: the different schools of rehabilitation

Given our knowledge of eye movement and visual perception psychophysics and physiology and of the different ways visual systems may be injured, cognitive neuropsychology has enabled researchers to basically examine separately cortical vs. subcortical pathways of vision, and to clinically develop tools to rehabilitate impaired visual functions and/or boost spared ones. For a review on principles underlying visual training programs, see in the present e-book the article by Taub et al. (2014). Since the late 1970s and early 1980s, different schools have emerged in Europe and USA, which we now briefly review.

Recovery of some residual capabilities in patients suffering from organic lesion of one or several visual pathways (so-called neurovisual disorders) has been well documented. Pöppel et al. (1973) were the first to report that a patient suffering from a right HH following a left retrochiasmatic lesion was nevertheless able to accurately saccade to a flash presented in his blind visual field, though he had no conscious experience of his performance. Weiskrantz et al. (1974) called “blindsight” the ability of HH patients to perform well in visual tasks though they have no conscious experience of such performance. Blindsight includes eye movement abilities but also pointing, reaching and prehension tasks, identification of one of two objects or discrimination between shape and color attributes in forced-choice tasks (Weiskrantz et al., 1974).Whether blindsight is due to either spared cortical areas or functional subcortical routes is still under debate. For a review on blindsight, see in the present e-book the article by Perez and Chokron (2014). Consistent with the subcortical hypothesis, particularly the retino-collicular route linked to limbic system, HH patients can discriminate between fearful vs. neutral faces presented in their blind visual field, again without any consciousness of their performance, indicating that blindsight extends to affective performance (de Gelder et al., 1999). The first evidence of the involvement of the subcortical visual pathway in HH was brought by Sahraie et al. (1997). In this study, fMRI evidenced the involvement of the SC in HH patient GY performing a task well in which he had to decide the direction of a stimulus presented in his blind visual field. Additionally, Morris et al. (2001) invited patient GY to be presented with fearful vs. happy faces in his blind visual field. Using PET, the authors evidenced that fearful faces activated the neural network involving the retino-collicular visual pathway and the amygdala, providing further direct evidence that the colliculo-thalamo-amygdala complex is associated with unconscious visual perception of fearful face stimuli.

Rehabilitative techniques and methods in visual neuropsychology have been first initiated in Germany, USA, and France. In Germany, Zihl has developed visual training based on reading, pointing and eye movements (Zihl, 1980, 1995a,b). In this training, HH patients are invited to read a text moving on a screen, to point towards visual targets, and to make auditory saccades at different locations in space. Such training has been evidenced to enlarge the visual field as assessed by perimetry and to improve saccadic behavior. Based on this research, Kasten and Sabel introduced a standardized visual training so-called “Visual Restoration Therapy” (VRT, NovaVision© ) (Kasten and Sabel, 1995). In VRT, home-training of the blind visual field using computer-controlled stimuli allows patients suffering from HH or more restricted visual field defects to significantly enlarge their visual field size (Sabel et al., 2004; Jobke et al., 2009). Such improvement in vision seems to be independent of eye movements (Kasten et al., 2006) though it remains under debate (Horton, 2005). In line with these studies, Schuett (2009) has demonstrated the benefits of such rehabilitation in hemianopic dyslexia.

In USA, based on the observation that right traumatic brain injury patients exhibited deficits in visual exploration and in attentional span size, Ben-Yishay and Diller (1981, 1993) have developped visual training focused on attention. Specifically, the authors have used reinforcement of selective attention to improve visual exploration on the one hand, and of working memory, particulary central executive (the attentional control part of working memory), to boost planning, self-efficacy and consciousness on the other. This pioneer research has influenced visual entrainments focusing on executive functioning to rehabilitate perceptual deficits (e.g., Blázquez-Alisente et al., 2004).

In UK, since the seminal work by Weiskrantz et al. (1974) mentioned above, new tools have been discovered to assess the blind visual field of neurovisual patients. The latest one is the blindfield pupil response, which is attenuated in amplitude as compared to that of the sighted field and may be a predictor of intact psychophysical capacity in cortical visual field defects (Sahraie et al., 2013). On the other hand, Kennard and Pampakian have studied in HH the impact of saccadic eye movements in visual trainings. They have showed that saccades are improved with visual training, but eye movements may be more the effect than the cause of visual improvement (Pambakian and Kennard, 1997; Pambakian et al., 2004; Mannan et al., 2010). Additionally and consistent with Sahraie’s pioneer research (Sahraie et al., 1997), the English school has been putting emphasis on the retino-collicular route to account for blindsight capabilities, particularly through its links with limbic system (Tamietto et al., 2009, 2010, 2012). On a pragmatic note, Leff et al. (Koiava et al., 2012; Ong et al., 2012) have proposed Read-Right©, a web application for diagnosing HH and rehabilitating hemianopic alexia.

In France, Ducarne, Bergego, and Barbeau were the first to develop visual neuropsychology in brain-damaged children and adults (Ducarne and Barbeau, 1981; Ducarne et al., 1981, 1983). Based on their research, they formalized assessment and rehabilitation protocols of visual field defects and neglect (Barbeau, 1992; Ducarne De Ribaucourt and Barbeau, 1993; Vital-Durand and Barbeau, 1995). In this visual training program, rehabilitation is organized around somatognosic (body, posture) and proprioceptive processes, reinforcement of visual afferences through acoustic and tactile afferences, residual visual capabilities using light, movement and colors, cognitive processes involving extrabody space representations, and verbal instructions. Special interest is also given to eye movements through the training of visual alert and smooth pursuit. Technically, reinforcement involves one-target or two or more targets’ stimuli in tactile, proprioceptive, acoustic, manual and lower limb (leg, foot) modalities in experimental, pragmatic, and visuo-constructional situations (Ducarne De Ribaucourt and Barbeau, 1993). In line with this work, Chokron et al. showed in HH enlargement of contralesional visual field between pre- and post-training periods as assessed by automated perimetry (Chokron et al., 2008). Using fMRI and putting emphasis on retino-occipital route, these authors have also reported differential reorganization of visual cortical areas depending on lesion hemispheric side (Perez et al., 2009, 2013). For a review on functional brain imaging of visual training programs, see in the present e-book the article by Urbanski et al. (2014).

To end, it is worth noting that new technologies have also revolutionized visual training programs. As examples, repetitive transcranial magnetic stimulation (rTMS) and transcranial direct current stimulation (tDCS) have emerged as useful techniques to boost cortical areas in the neighborhood of lesioned occipital cortex and indirectly neuromodulate visual capabilities (Valero-Cabré et al., 2011; Brunoni et al., 2012; Afifi et al., 2013). For a review on non-invasive manipulation of frontal regions and eye movements, see in the present e-book the article by Vernet et al. (2014). Furthermore, Amedi et al. have shown that so-called visual areas (e.g., EBA) may not be visual but supramodal as evidenced by functional brain imaging of congenitally blind patients (Striem-Amit and Amedi, 2014). Indeed these areas can support sensory substitution and allow blind patients to see through non-visual information like sounds or music (Maidenbaum et al., 2014; Proulx et al., 2014).

To summarize, various visual training programs have been developped since the 1970s in USA and Europe based on psychophysical, functional brain imaging, neuromodulation and substitution discoveries. Depending on theoretical frameworks, trainings have focused on action (the French school), on perception (the German and English schools), or on attention (the American school), resulting in each case in improvement of both eye movement behavior and visual perception.

## Conclusion

Making ours von Goethe’s percept (Von Goethe, 1808–1832/2014), we have emphasized in this review that vision is first and foremost action. The variety and subtlety of eye movements (saccades, pursuit, vergences, etc.) require the perfect orchestration of a cascade of physiological mechanisms from motoneurons to cortical areas. This movement machinery not only ensures binocular coordination to foveate targets at any location in 3D space, but is also a prerequisite for visual perception since action precedes perception. We are aware that many actions may be based on the preceding perceptual information, or in a stabilization context after removing movement there is still perception before the visual input fades. But because movement is essentially unavoidable (e.g., oculomotor tremor, drifts, head/body movements) and desirable for exploration, movement has always existed in animals and the evolution of perception has always dealt with this reality. In that sense movement has always preceded the evolution of our perception. In this context, we suggest that “visual action” is a more physiologically plausible expression than “active vision”, which appears to be in most cases a pleonasm. Visual perception is achieved through five main systems and, importantly, any visual stimulus activates these different pathways. The retino-occipital and retino-collicular routes are widely studied, whereas the retino-pretectal, the retino-hypothalamic, and the AOS are less explored. Therefore, our knowledge of complete or partial visual field defects inherent in antero- or retro-chiasmatic lesions, and that of high-level visual deficits (agnosias and visual action disorders) following a lesion of ventral or dorsal streams is well advanced. But studies exploring the impact of visual disorders on pupillary response, rhythms, biological functions (e.g., sleep or hormonal disorders), or on slow movements are lacking. Consistent with the neural bases of eye movements and visual pathways, action-perception integrated and multimodal interventions seem to provide the best results in visual rehabilitation. This suggests that any rehabilitative training in neuropsychology should first take into account the cognitive and cerebral constraints. In other words, any training should fit the physiology in its resources, plasticity, and limitations. Because vision recruits 60% of the brain (Orban et al., 2004; Orban, 2007), eye movements and visual perception are useful tools to assess and rehabilitate the CNS in general. In other words, vision in its motor and perceptual aspects may be useful biomarker and neuromodulator of CNS functioning: education in children and in normal aging, rehabilitation in CNS functional disorders, in neurological and psychiatric diseases, and in pathological aging. For an original research on eye movements and visual perception in Alzheimer’s disease, see in the present e-book the article by Boucart et al. (2014). For a review on visual perception in schizophrenia, see in the present e-book the article by Notredame et al. (2014). For an original research on eye movements in bipolar disorder, see in the present e-book the article by Beynel et al. (2014). With respect to functional disorders, recent findings have suggested that visual disorders hitherto supposed to be peripheral may have cerebral causes and/or effects. This might be the case of anisometropia, amblyopia, strabismus, which involve CNS dysfunctioning as revealed by neurophysiological studies in animals and functional brain imaging studies in humans. For a review on functional brain imaging of amblyopia, see in the present e-book the article by Joly and Frankó (2014). For an original research on eye movements in amblyopia, see in the present e-book the article by Perdziak et al. (2014). For a review on neurophysiology of amblyopia, see in the present e-book the article by Bui Quoc and Milleret (2014). Thus vision in its complexity and richness offers exciting directions for future basic and clinical research.

## Conflict of interest statement

The authors declare that the research was conducted in the absence of any commercial or financial relationships that could be construed as a potential conflict of interest.
